# Male DCIS diagnosed after use of over-the-counter hormonal supplement

**DOI:** 10.1016/j.ijscr.2019.02.028

**Published:** 2019-02-26

**Authors:** S.O. Agbroko, K.E. Rojas, D.M. Manasseh, P. Borgen

**Affiliations:** a Department of Obstetrics and Gynecology, Maimonides Medical Center, Brooklyn NY, United States; b Department of Surgery, Maimonides Medical Center, Brooklyn NY, United States

**Keywords:** Ductal carcinoma in-situ, Male breast cancer, Mastectomy, Gynecomastia, Atypical ductal hyperplasia

## Abstract

**Introduction:**

Male breast cancer is a rare disease. Although epidemiologic and genetic factors are associated with male breast cancer, hormonal factors may also play a role.

**Case presentation:**

We report the case of a 39-year-old BRCA negative male patient taking a sexual performance enhancement supplement who presented with worsening asymmetric gynecomastia and unilateral spontaneous bloody nipple discharge and was found to have ductal carcinoma in-situ.

**Discussion:**

The altered cellular environment related to the hormone contents of the supplement coincided with the rapid worsening of his gynecomastia and may have played a role in the development of the ductal carcinoma in-situ, or growth of an existing focus.

**Conclusion:**

The use of hormonal male enhancement supplements can lead to higher levels of androgens in users. It is possible for this altered hormonal environment to cause the growth of tumor or promote the progression of an existing focus.

## Highlights

• As illustrated in this case report, male ductal carcinoma in-situ is a rare disease, and treatment is not standardized.
• Hormone-containing male enhancement supplements are unregulated and available over-the-counter.
• Hormonal supplementation may be related to the development or worsening of rare cases of male breast cancer.

## 1 Introduction

Male breast cancer comprises less than 1% of all breast cancer and less than 0.1% of all cancer-related mortality in males [1]. While an increase in the uptake of population-wide screening for female breast cancer has led to a larger proportion of early stage diagnoses in women, there are no specific screening guidelines for men. Limited awareness of male breast cancer both by patients and providers may also lead to later stage at presentation and oftentimes worse prognosis.

In both women and men, the most common cause of a heritable increase in breast cancer susceptibility is due to pathogenic mutations in the BRCA genes associated with DNA repair [2]. With regards to environmental risk factors, most male breast cancers (like female breast cancers) express the estrogen receptor. Therefore, any disease condition or exogenous supplementation that causes an increase in circulating estrogens may also increase the risk of developing breast cancer. While the Women’s Health Initiative (WHI) study demonstrated that long-term use of combined estrogen and progesterone non-significantly increased the risk of breast cancer in women, the potentially tumorigenic effects in men have not been completely described on a population-wide basis [3]. Furthermore, obesity and its associated metabolic syndrome may lead to a hormonal profile that favors epithelial proliferation in estrogen-sensitive tissues like the breast. The use of hormonal supplements in males may also cause an environment conducive for the development of malignancy. In this case report we explore the possible association of male hormonal supplements with the development of male breast cancer.

This case report has been conducted in line with the SCARE criteria [4].

## 2 Presentation of case

A 39-year-old Sudanese male presented to an urban nationally-accredited comprehensive Breast Health Center with a longstanding history of bilateral gynecomastia and new onset unilateral bloody nipple discharge. He denied recent infection or trauma. When asked about medication use, he revealed that he had started daily use of an over-the-counter herbal male enhancement supplement several months prior and had since noticed asymmetric breast enlargement.

**Fig. 1:**
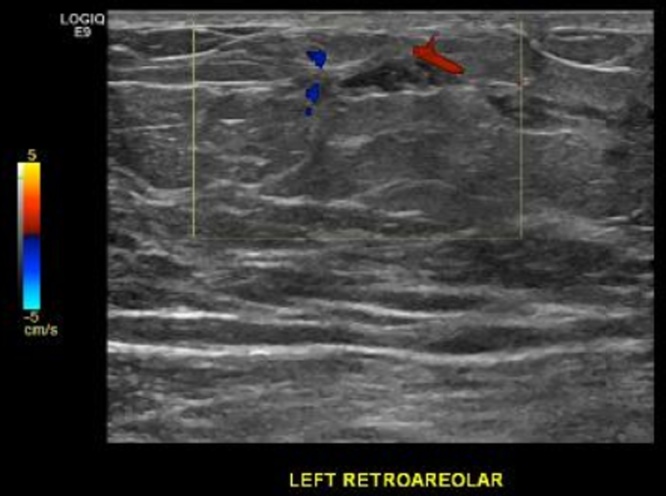
Ultrasound image of left retroareolar mass.

**Fig. 2:**
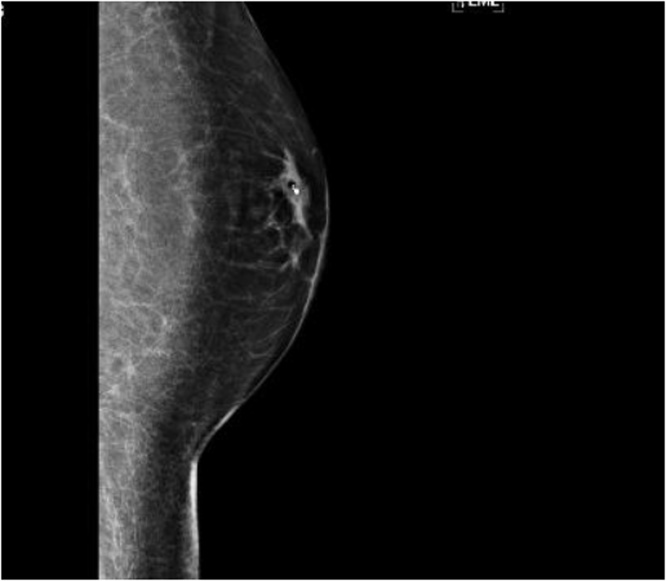
Left breast digital mammogram, ML view with biopsy clip.

Review of his prior medical and surgical history was otherwise noncontributory. Furthermore, he had no significant family history of breast or ovarian cancer. He did report that other male family members also had gynecomastia. Physical examination was remarkable for bilateral asymmetric gynecomastia, left greater than right. He was found to have a non-discrete retro areolar mobile mass measuring approximately 1 × 1 cm. Of note, the patient was overweight with a BMI of 27.5. Bilateral mammogram and ultrasound revealed dilated ducts in the left retroareolar region without an apparent intraductal mass, and further imaging was recommended (Fig. 1, Fig. 2). Contrast-enhanced MRI (3T) using a breast-specific protocol demonstrated a left periareolar non-enhancing mass, and ultrasound-guided core biopsy using a 14-Gauge automatic device showed atypical ductal hyperplasia with calcifications.

The pathology results were discussed with the patient and the potential for discordance in the presence of a mass was disclosed. The decision was made to proceed with wire-localized excision of the left breast lesion to completely characterize the pathology. The lumpectomy surgical pathology revealed ductal carcinoma in-situ (DCIS) of intermediate nuclear grade with papillary and cribriform types. DCIS was present focally at the anterior and lateral margins and within 1 mm of the superior and inferior margins. Notably, the lesion was both estrogen and progesterone receptor positive

The option of re-excision versus mastectomy was discussed with the patient who elected to undergo genetic counselling prior to making a decision. He was referred for expedited genetic counselling and tested negative for a BRCA1 and BRCA2 pathologic mutation. In spite of the negative results, the patient elected to undergo bilateral mastectomy for simultaneous treatment of the lesion and the preexisting bothersome gynecomastia. The right breast was eligible for nipple-sparing mastectomy but given the retroareolar location of the left breast lesion, the nipple areolar complex was unable to be spared. The patient met with plastic surgery and decided to proceed with delayed left nipple reconstruction to eventually achieve the most symmetric outcome possible.

Left mastectomy pathology revealed a minute focus (<1 mm) of DCIS of intermediate grade and the margins were widely uninvolved. A single sentinel lymph node was negative for malignancy and the right breast contained only benign findings.

## 3 Discussion

In summary, a 39-year-old Sudanese male presented with worsening asymmetric bilateral gynecomastia and new onset bloody left nipple discharge in the setting of recent initiation of an androgen-containing supplement. He was found to have unilateral estrogen-sensitive DCIS for which he underwent bilateral mastectomy. Despite being a young male with breast cancer, there was no genetic explanation for this patient’s malignancy. Although the possibility of harboring a not-yet-discovered oncogenic mutation does exist, his breast cancer is still considered sporadic.

Although causality could not be proven in this single case, obesity and exogenous hormone supplementation could be related to the development of his hormone-sensitive neoplasia. The Extenze Maximum Strength Male Enhancement Liquid Gelcaps (Biotrab Nutraceuticals) is an over-the-counter male enhancement supplement marketed as helping to “enhance pleasure and performance”. Review of the ingredient list revealed 50 mg of dehydroepiandrosterone sulphate (DHEAS) in each pill, and the suggested use is one capsule every morning. To our knowledge, there are no studies demonstrating an increased risk of breast cancer with exogenous androgen supplementation. However, peripheral aromatization of exogenous androgens could theoretically increase the circulating levels of estrogens in an overweight patient with significant adiposity [5]. Hormonal supplementation in women is largely in the form of hormone replacement therapy with estrogens and not androgens. In obese postmenopausal women, peripheral conversion of androgens to estrogen by aromatase underlies the hypersetrogenic state. It also underlies the higher incidence of hormone dependent malignancies like breast cancer in this population.

Notably, our patient also reported worsening gynecomastia with the ingestion of the male enhancement supplement. Gynecomastia affects 50–60% of adolescent males, and usually resolves with adulthood [6]. Although more often considered a cosmetic rather than an oncologic problem, it has been found to be present in 6–38% of breast cancer cases in men [7,8]. In a series of 104 patients managed between 1975 and 1990 by Borgen and colleagues, 23% of patients with breast cancer had gynecomastia, with DCIS constituting 15% of the malignant diagnoses [9].

Treatment of males with breast cancer is based upon protocols extrapolated from studies of female breast cancer and usually consists of surgery, adjuvant chemotherapy and/or endocrine therapy and potentially adjuvant radiation depending on surgical modality and stage at presentation. These treatment options have proven to be equally effective in males as in females [[10], [11], [12]]. Although survival outcomes are similar between male and female breast cancer patients when matched by stage, most men present later in the course of their disease. While approximately 2/3 of women diagnosed with breast cancer are found to have DCIS or Stage 1 invasive breast cancer, more than 2/3 of male breast diagnoses are Stage 2 or greater [[13], [14], [15], [16]].

The rarity of male ductal carcinoma in-situ, along with our patient’s young age and lack of a known pathogenic mutation also make this case notable. Furthermore, it is unclear if the exogenous “male enhancement” supplementation was tumorigenic in itself or may have led to the conversion of a minute atypical focus of breast tissue into in-situ carcinoma. Nevertheless, the timing of worsening asymmetric gynecomastia and new pathologic nipple discharge in relation to the administration of the supplement are concerning. In contrast to the assessment of trial-proven pharmaceuticals, the regulatory approach of the Food and Drug Administration with regards to over-the-counter supplements is that they are “safe until proven unsafe” [17]. Although the potentially carcinogenic effects of exogenous hormonal supplementation have been described in the literature, consumers are able to purchase unregulated hormonal derivatives without the supervision of a medical professional. Improved regulation of the availability of these products, along with addressing consumer misinformation about their safety should be addressed in order to minimize potential harm to end-users [17].

## 4 Conclusion

The use of androgenic male hormonal supplements could lead to a hormonal environment conducive for the development of breast cancer. While there is no clear proof of causation, the conversion of male hormones to estrogens in the peripheral tissues can lead to a hyperestrogenic environment favorable to the development of estrogen dependent and sensitive tumors. This risk should be borne in mind by consumers of supplements with hormonal ingredients.

## Conflict of interest

There is no conflict of interest.

## Sources of funding

There was no funding for this case report.

## Ethical approval

This was a single case report and IRB approval was not required.

## Consent

Patient provided a written informed consent for the publication of this case report.

## Author contribution

Dr Agbroko did the relevant literature search and development of the case report.

Dr Rojas provided repeated edits of the write up and also guided the development of the report.

Dr Manasseh was the primary surgeon and provided oversight for this work.

Dr Borgen also provided editorial inputs and supervised the work.

## Registration of research studies

Not applicable to this case report.

## Guarantor

Dr Kristin Rojas.

## Provenance and peer review

Not commissioned, externally peer-reviewed.
